# Is There a Burnout Epidemic among Medical Students?

**DOI:** 10.1192/j.eurpsy.2025.86

**Published:** 2025-08-26

**Authors:** M. Di Vincenzo

**Affiliations:** Department of Psychiatry, University of Campania “Luigi Vanvitelli”, Naples, Italy

## Abstract

**Abstract:**

Medical students are persistently exposed to specific conditions that may have detrimental effects on mental health, such as stressful academic routine, demanding and heterogeneous curriculum, sense of competition and daily exposure to illness and death. Accumulating evidence highlights high levels of cynicism and emotional exhaustion among these subjects. Indeed, the prevalence of burnout syndrome in medical students may reach peaks up to 88% in some studies. Thoughts of stopping medical education, negative life events, lack of support, dissatisfaction, and poor motivation represent the main predictors of burnout in this group. Such findings highlight the need to develop prevention initiatives targeting the future generation of medical doctors. Moreover, implementation of psychosocial interventions delivered from medical schools could be helpful to improve coping strategies and resilience styles of students.

**Disclosure of Interest:**

None Declared

